# Nanocrystalline Silver for the Treatment of Otomycosis: A Retrospective Study

**DOI:** 10.22038/IJORL.2023.66805.3303

**Published:** 2023-03

**Authors:** Aditi Sambhaji Moruskar, Vinod Shinde, Mayur H. Ingale, Arpita A. Krishna, Rishikesh D. Pawar

**Affiliations:** 1 *Department of Otorhinolaryngology, Dr. D.Y. Patil Medical College and Hospital, Dr. D. Y. Patil Vidyapeeth, Pimpri, Pune, India.*

**Keywords:** Nanocrystalline silver, Otomycosis.

## Abstract

**Introduction:**

Otomycosis is a common fungal ear infection usually found in tropical and subtropical countries where infections arise due to hot and humid conditions. Also, these infections have a high recurrence rate with limited therapeutic options, which makes their management difficult. There is a long history of applying various antiseptic agents consisting of silver specifically for these broad-spectrum infections. Silver nanoparticles (AgNPs) are futuristic nano-size products for controlling the microbial infection. The study aimed to determine the antifungal properties of nanocrystalline silver in patients with otomycosis.

**Materials and Methods:**

The study was conducted in Pune (India) in the department of Ear Nose Throat & Head at the Dr. D.Y. Patil Medical College, Hospital and research center, Pune (India) for a period of one year (2019 -2020). Our study included 100 patients (58 male and 42 female) with clinically diagnosed otomycosis, which was treated by applying nanocrystalline silver gel-soaked Gelfoam.

**Results:**

Our study had patients of 18 to 60 years, with the highest prevalence in males (58%) aged 30 to 45 years. A large number of infection cases were reported at the hospital, i.e., 62 cases during the wet season as compared to 38 cases during the dry season. Commonly found fungi belonging to the genus *Aspergillus* (55%), followed by *Candida* (45%). Improvements in symptoms were observed in 89% of the patients (70% within 5 to 6 days and 19% from 7 to 14 days).

**Conclusion:**

Applying nanocrystalline silver cured most of the patients (89%) within 14 days. Treating otomycosis patients with nanocrystalline silver demonstrated beneficial results. Further studies with larger samples should be conducted to validate the benefits of nanocrystalline silver.

## Introduction

Otomycosis is a superficial fungal infection generally observed in the medial facet of the external auditory canal (EAC) caused by opportunistic fungi (1). It is a common medical issue encountered by ENT surgeons in India, mainly due to its tropical wet climate condition (2). It is a presenting problem in 6-9% of otologic patients in tropical countries like Nepal (3,4). It is also challenging to manage both patients and clinicians due to the limited therapeutic options (5). 

Past reported cases had observed complications involving the inner ear, and in rare cases, mortality was also seen (5). The infection is characterized by itchiness, disturbance to the EAC, scaling and severe uneasiness with agony, and in some cases, hypoacusis (1,5). 

Otomycosis occurs worldwide, but it is predominant mostly in tropical, tropical-wet, and sub-tropical countries having favorable conditions such as frequent heat and high levels of humidity, which assists in fungal growth (5,6). According to past reports, there are five to 25% of otomycosis cases from tropical countries concluded with otitis externa (3).

There are various predisposing factors for otomycosis which include failure in the natural defense mechanisms of the ear such as deviations in the covering thin layer of epithelium, favorable pH conditions, varying amount and thickness of ear wax, and assisting bacterial infection. Also, among the several causes of otomycosis are self-administered trauma such as applying hearing aids or prostheses, using Q-tips for cleaning the ears, ingestion of antibiotics, steroids, etc., and neoplasia (7- 9).

The outcomes seen due to otomycosis involve debris collecting in the ear contaminated with fungi, exfoliation of the superficial thin epithelial layer, inflammation of EAC skin, and pus formation without any destruction of the tympanic membrane. The presence of greyish-white or black thick debris or cheese-like tissues is the most frequent finding during examination (10-12). 

Otomycosis is widespread among adults but also observed among different age groups as per the predisposing factors discussed above. The prevalence is higher in the mid-twenties and lower in teenagers and people over the age of 60 years (12-14). Our observations contradict those reporting a higher prevalence among women (13,15).

Treatment recommendations include careful cleaning of the EAC under the operating microscope and proper identification of causal micro-organisms, their elimination or limited exposure to the predisposing factors, microbial aspiration, and sometimes, using antifungal agents (3,7,16). Antifungal drops should be used for a minimum of three weeks to prevent the recurrence of infection and it may be continued even after symptomatic relief. The necessary prerequisite for a long-lasting cure is restoring the specific environment of the bottom part of the EAC (4).

The most common strains causing otomycosis are *Aspergillus* and *Candida *species. Their predominance may vary based on their geographical locations (9,11,13). These fungi can cause opportunistic infections and constitute broad-spectrum pathogenicity based on the infected strain by infusing the normal microbiota of the host body parts (17,18). Common infecting yeast such as *Candida* *albicans* and *Candida tropicalis* are mostly responsible for numerous human diseases. The excessive use of antibiotics against fungal infections results in increased resistant strains among otomycosis. Hence, these issues have initiated new research to find an effective and alternative way to eradicate these infections (19).

Nanotechnology is a boon in recent times responsible for producing novel metal nanoparticles. These nanoparticles specifically target the infected micro-organisms which are potent antimicrobial tools for eradicating these micro-organisms which are resistant to conventional drugs (20). Past research by DC Gheorge showed that nanoparticle-based systems outperform the conventional drug therapy approach as it is more specific and stringent in action (20). 

Nanoparticles specifically target the micro-organism and transport the active substance preparations to the desired site *via* inter-membrane transportation, as the cell easily uptakes the nano size with minimal dose requirement and without causing any side effects (21). According to past reports, silver is a key component of conventional anti-fungal agents, showing strong inhibitory and antimicrobial effects. It has higher toxicity towards microbes as compared to other heavy metals though it demonstrates limited toxicity to mammalian cells. Bio-stabilizing anti-fungal coatings use silver nanoparticles (AgNPs) (19,22,23). In cases of otomycosis, coating the nanoparticles with silver nitrate gel has beneficial effects (24). 

## Materials and Methods

*Study design:*

The present study was a retrospective one constituting 100 patients of 18 to 60 years was included from July 2019 to June 2020. The study was conducted in the outpatient clinic of the department of Ear, Nose, Throat & Head at Dr. D. Y. Patil Medical College, Hospital and Research center, DPU. Following clinical details were noted such as name, age, gender, address, occupation, chief complaint, any history of infection, suspected risk factors, predisposing conditions, ear affected, species of fungus, and antifungal agent applied. Approval from the Ethics committee was taken and informed written consent was obtained from all the patients. Inclusion criteria - Patients with the clinical diagnosis of otomycosis confirmed *via* otoscopy with an intact tympanic membrane between 18-60 years. Exclusion criteria – Patients with tympanic membrane perforation, active middle ear infection, attic retraction pocket, cholesteatoma, and operated cases of canal wall down mastoidectomy. 

*Procedure*

Patients who complained of itching, blocking sensation in the ear, hearing loss, pain, and sticky discharge were examined with an otoscope. White, brown, or black fungal mass was observed. Then, they were subjected to ear microscopy. The external ear canal’s outer part was cleansed with sterile cotton stretched over a Jobson Horne probe. A sterile cotton swab was gently rotated after being placed deep into the canal to collect fungal hyphae and discharge. The swab was sent to the microbiology section in a sterile culture tube. The samples were then processed by direct slide microscopy using KOH and cultured in Sabouraud Chloramphenicol agar for 3-4 weeks, followed by weekly observation. Once growth was seen on agar plates, direct microscopy with Lactophenol cotton blue stain was applied to identify fungal morphology. All potential debris and discharge were removed after the canal’s fungus mass was suction cleaned. Myringitis and superficial epithelial exfoliation of canal skin were observed as signs of inflammation. Gelfoam was fragmented and soaked in nanocrystalline gel (Nanomac Silver gel). Large sterile cotton was stored in the concha, and the canal was entirely filled with Gelfoam pieces. Patients were requested to follow up every 5^th ^day. On the 5^th^ day, cotton from the concha was removed. Gelfoam pieces in the EAC were removed. Any residual inflammation-related symptoms were checked in the tympanic membrane and EAC. After five days, patients who still had inflammation underwent another filling of the EAC with Gelfoam dipped in the nanocrystalline gel. Patients were requested again to follow up after five days and Gelfoam pieces were removed from the EAC. Finally, after 14 days, conventional antifungal treatment with antifungal ear drops and oral antibiotics were started for those patients who still had inflammation.

*Statistical analysis*

Mid-P was calculated by applying Winpepi software.

## Results

A total of 100 confirmed clinical diagnosis cases of otomycosis were observed by direct microscopic examination. The age of the patients ranged from 16 to 60 years. The study group constituted mostly patients in the age group of 30-45 years (64%). Out of the 100 patients, 58 were male, and 42 were female (Table 1). We observed 45% of patients with right ear infections, 40% with left ear infections, and 15% cases with bilateral infections (Table 2). As the percentage found was the same as the absolute number, hence, it was not mentioned.

**Table 1:** Gender-wise distribution of etiological fungal species

**FungalSpecies**	**Total no. of patients**	**Male patients**	**Female patients**
Aspergillus flavus	47	27	20
Candida albicans	31	16	15
Aspergillus niger	17	12	5
Aspergillus fumigatus	5	3	2
Total	100	58	42

**Table 2:** Distribution of fungal species by the ear affected

**Fungal Species** **(No. of patients)**	**Ear affected**		
	**Right Ear**	**Left ear**	**Bilateral ear**
Aspergillus flavus (47)	17	17	13
Candida albicans (31)	15	15	1
Aspergillus niger (17)	10	6	1
Aspergillus fumigatus (5)	3	2	0
Total	45	40	15

**Fig 1:**
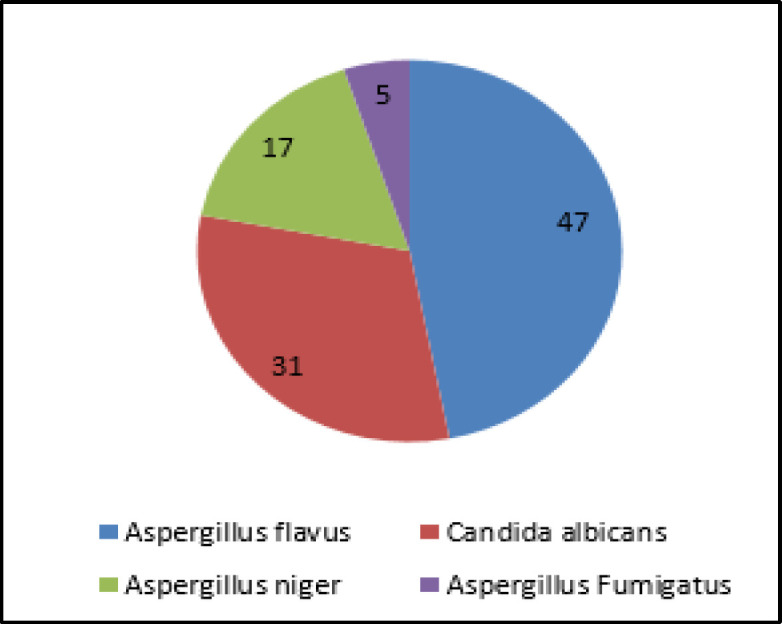
Fungal species-wise distribution of patients (absolute numbers)

The most frequent fungal infections were observed from the *Aspergillus* genus (69%), followed by *Candida* genus (31%). Other species identified were *A. flavus (*47%), *C. albicans* (31%), *A. niger* (17%), and 5% *A. fumigates* (5%) (Figure 1).

The included cases were divided into three groups based on the clinical response to the treatment. These groups are as follows: early responders to treatment (having clear or dry EAC and the tympanic membrane in the absence of secretion), partial responders to treatment (minor discharge but the ear is not completely dry), and non-responders to treatment (having discharge in EAC, exfoliation of EAC skin, myringitis). The treatment was discontinued in patients of the first group while other groups were given treatment. After 14^th^ day, if the discharge still continued, then the case was considered as treatment-resistant and hence, further treated with conventional antifungal ear drops and oral antibiotics. 

An equal number of patients were affected on the right and left ears with *Aspergillus flavus* (17 each) and *Candida albicans* (15 each). Patients of age 30 to 45 years were most affected by *Aspergillus flavus* as compared to other age groups. But, patients of 45 to 60 years of age were more commonly infected with *Candida albicans* as compared to other age groups (Table 3).

**Table 3:** Distribution of fungal species based on patients’ age group

**Fungal Species (No. of patients)**	**Age in years – Total no. of patients**		
	**18-30 Years**	**30-45 Years**	**45-60 Years**
Aspergillus flavus (47)	12	25	10
Candida albicans (31)	4	13	14
Aspergillus niger (17)	4	5	8
Aspergillus fumigatus (5)	0	1	4
Total	20	44	36

**Table 4:** Distribution of fungal species by seasonal variation

**Fungal Species**	**Total no. of patients**	**Wet Season**	**Dry Season**
Aspergillus flavus	47	30	17
Candida albicans	31	20	11
Aspergillus niger	17	8	9
Aspergillus fumigatus	5	4	1
Total	100	62	38

More patients were affected during the wet season as compared to the dry season by *Aspergillus flavus* and *Candida albicans*.

**Table 5:** Duration-wise response to treatment

**Duration**	**Symptomatically improved no. of patients (95% Confidence Interval)**
5-6 days	70 (60.5- 78.4)
7-14 days	19 (12.2-27.6)
No significant improvement after 2 weeks	11 (5.9-18.3)

Less than one-fifth of patients improved between seven and fourteen days, compared to less than three-quarters (70%) who did so within five to six days. In total, 89% of people experienced symptomatic relief after 14 days (Table 6). 

**Table 6:** Duration-wise symptomatic improvement of fungal species types

**Fungal Species**	**Total no. of patients**	**Symptomatically improved no. of patients**		
		**5-6 days (%)**	**7-14 days**	**No improvement at 2 weeks**
Aspergillus flavus	47	33 (70.21%)	10 (21.27%)	4 (8.51%)
Candida albicans	31	25 (80.64%)	4 (12.90%)	2 (6.45%)
Aspergillus niger	17	9 (52.94%)	4 (23.52%)	4 (23.52%)
Aspergillus fumigatus	5	3 (60%)	1 (20%)	1 (20%)
Total	100	70 (70%)	19 (19%)	11 (11%)

**Fig 2:**
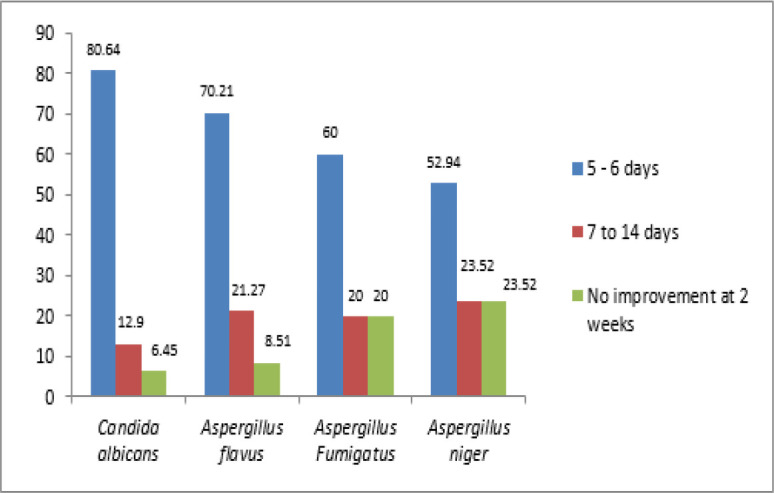
Types of fungal species by duration-wise symptomatic improvement

In all cases with different fungal species, a higher number of patients showed improvement in five days (Figure 2).

## Discussion

Otomycoses are frequent fungal infections in tropical, tropical wet, and sub-tropical countries because of favorable heat and humid conditions (5). Usually, otomycosis can be diagnosed on clinical examination in an Out Patient Department (OPD) setup. The most frequent symptoms observed are otalgia, pruritus, otorrhea, and hypoacusis (18). 

Based on the findings, the incidence rates of bilateral otomycosis were very low (3). Ho *et al*. (2006) reported bilateral cases of the ear in only 7% of the study group, while our study observed 15% of bilateral cases (10). 

There were predominantly male patients (58%) who were more commonly affected by otomycosis than female patients (42%) in our study. These findings are similar to results observed by Kaur *et al*. (2000) (18), Ho *et al.* (2006) ^(^^10^^)^, and Yehia *et al.* (1990) (6), who reported 60%, 56%, and 52.5% cases of otomycosis in males, respectively. But contradictory results were also observed by Pontes *et al.* (2009) and Zaror *et al.* (1991) (9) (25), whose findings suggest a predominance among females i.e., 60% and 65% cases, respectively. Other studies reported no significant difference between genders for otomycosis susceptibility (Keyvan *et al.* 2018) (26). However, the affected person typically belongs to the category of people who work outside the home, which is a common factor across all studies. 

Out of the total cases, 20% of the cases were found in the age group of 18-30 years, and 44% of cases were in the age group of 30-45 years. But, Pontes *et al.* found 60% of cases were between 2 to 20 years of age (9).

Reports by Prasad *et al*. observed an increase in the number of otomycosis cases (78%) in the rainy season compared to other months (22%) of the year in Mangalore (14), India, which clearly justified that the wet environment induces the growth and transmittance of fungal infection.

The most common infectious fungal strains were *Aspergillus* and *Candida*. Various studies reported a greater prevalence of *Aspergillus *(*niger, flavus,* and other spp.) as otomycosis agents (5,18,27). Our data coincided with this observation where 69% of *Aspergillus* spp. (*niger*, *flavus*, and *fumigatus*) and 31% of isolates of *Candida* spp. (*albicans*) were found in the cases. A similar observation was also reported by Keyvan *et al.* (2018)(26), where *A*. *flavus* was the common infectious agent (63.33%), and *Candida *only caused otomycosis in 12.66% out of total cases. On contrary, Jaiswal *et al.* (1990) found 46% of *Candida *species (13). 

Banach *et al.* observed that the effective concentration of silver nanoparticles was 1-3% against fungal infection, which is equivalent to 5-15 ppm nanosilver (22). In our study, the nanocrystalline silver concentration in the gel used was 20 µg/1 gm. In a study by Hasselt P, a single dose of 1% silver nitrate gel cured 92% of ears with otitis externa (24).

Nanosilver exhibits strong fungicidal capabilities against *Candid*a spp. and on dermatophyte Trichophyton mentagrophytes, according to medical investigations. The effective concentration reported was 1 mg/dm^3^ (28). A similar study by Mallmann *et al.* reported high fungicidal activity of silver nanoparticles against *Candida* spp (*albicans* and *tropicalis*) (19). Ag-NPs also exhibited a lethal activity against *C. albicans* and *Saccharomyces cerevisiae (*Nasrollahi et al.) (29). In our study, we found 31 patients with *Candida* spp. infection, 25 patients (80.64%) had symptomatic improvement within 5-6 days after the nanocrystalline silver gel was applied. Out of 47 patients with *A. flavus* infection, 33 patients (70.21%) had symptomatic improvement within 5-6 days.

Of the 11 patients who had received treatment but had no response at 14 days, six (54.54%) were between the ages of 45-60, 4 (36.36%) were between the ages of 30-45 and one (9.09%) was between the ages group of 18-30. According to our study, 70% of otomycosis patients improved within 5–6 days, and 89% showed a significant improvement within two weeks.

*Limitation*

Little information is known on the antifungal characteristics of the silver nanocrystalline gel used to treat otomycosis. As a result, the assessment typically refers to or depends only on conventional research in this field. 

## Conclusion

The pilot study showed that otomycosis could be effectively treated with nanocrystalline silver gel. Wet or humid environmental conditions are conducive to the development of otomycosis, particularly in tropical areas. Considering that the symptoms of fungal infections are non-specific, clinical tracking of patients is essential. The benefits of nanocrystalline silver therapy on otomycosis patients require further study on larger samples in various geographic areas and during the wet and dry seasons in India.
